# LUMBER: A feasible MRI‐based 3D printed mould platform for ex vivo sampling of prostate cancer

**DOI:** 10.1002/bco2.70161

**Published:** 2026-02-24

**Authors:** Chris Du, Goran Rac, Nicholas Lanzotti, Jeffrey Ellis, Victor Chen, Maria M. Picken, Guliz A. Barkan, Michael Woods, Alex Gorbonos, Marcus Quek, Steven M. Shea, Gopal N. Gupta

**Affiliations:** ^1^ Department of Urology Loyola University Chicago Maywood Illinois USA; ^2^ Department of Urology Lexington Medical Center West Columbia South Carolina USA; ^3^ Department of Urology VA Boston Healthcare System Boston Massachusetts USA; ^4^ Department of Pathology Loyola University Chicago Maywood Illinois USA; ^5^ Radiology and Medical Imaging Loyola University Chicago Maywood Illinois USA; ^6^ Department of Surgery Loyola University Chicago Maywood Illinois USA

**Keywords:** 3D‐printing, cancerous tissue, ex vivo sampling, prostate cancer, prostate needle biopsy, prostatectomy

## Abstract

**Introduction:**

Accurate, reliable means to obtain fresh viable clinically localised prostate cancer tissue do not exist. We developed a method in which bespoke 3D‐printed moulds can be created for any prostate and allow for ex vivo sampling of magnetic resonance imaging (MRI)‐visible, biopsy‐proven cancer lesions. We sought to demonstrate the ability of our platform to obtain fresh viable prostate cancer tissue after robot‐assisted radical prostatectomy (RARP).

**Methods:**

Inclusion criteria were a patient that underwent MR‐fusion biopsy (UroNav, Philips) with a resulting biopsy proven Gleason Grade (GG) ≥ 2 target. STL files for prostate boundary and target regions of interest (ROI), created as part of fusion biopsy, were exported from the UroNav and imported into SolidWorks (Dassault), a solid modelling computer‐aided design and engineering application. A macro within SolidWorks was then applied to create a material‐optimised mould around the prostate with needle guides to allow for targeted sampling. The 3D mould was exported as an STL file and then 3D‐printed on a Stratasys Fortus 250 MC 3D Printer. During RARP, the specimen is extracted, the seminal vesicles detached and the prostate is placed in the mould for biopsy. The biopsy cores are sent to pathology for analysis and compared to specimens from the initial biopsy.

**Results:**

Twelve patients with MRI‐visible lesions and biopsy proven GGG ≥ 2 cancer underwent RARP. In 12 out of 12 patients, ex vivo biopsies performed with the 3D‐printed mould yielded prostate tissue with cancer.

**Conclusions:**

Our 3D‐printed mould platform allows for ex vivo sampling of MRI identified and previously biopsied prostate cancer at the time of RARP. The native, cancerous tissue may then be used to advance further research. The potential applications for a platform that can reliably sample living prostate cancer tissue are numerous, including the ability to advance future cancer research as well as other solid‐organ malignancies with targetable lesions.

## 1 INTRODUCTION

Globally, prostate cancer is the second most common and fifth leading cancer‐specific cause of death in men.^1^ After diagnosis, prostate cancer is risk‐stratified, and shared decision‐making is used to decide between various treatment modalities, including active surveillance, radical prostatectomy and radiation therapy.^2^ Nevertheless, more than half of patients (53%) with high‐risk prostate cancer will have disease recurrence, highlighting the need for continued advances.^3^

The tumour microenvironment plays a role in cancer survival after treatment, particularly in the prostate, where cancerous tissue is multifaceted, complex and of many cell types.^4^, ^5^ Having a variety of prostate cancer cell lines would greatly aid basic science research into new therapeutic treatments. However, prostate cancer tissue is not visibly different from normal prostate tissue, making it challenging to procure cancerous tissue for research while also providing necessary tissue for standard pathologic analysis. Furthermore, current pathological processing for prostate cancer involves fixing the specimen in formalin and then staining it in haematoxylin and eosin, resulting in non‐viable cancer tissue.^6^

The 3D printed moulds generated from existing MR prostate images have been used to improve the accuracy of comparing whole‐mount pathology slices from radical prostatectomy to MR image slices.^7^ This creates a feedback mechanism to radiologists for determining visual characteristics of prostate cancer in MR images.^8^ Registration results have also been used to train deep learning artificial intelligence models and to increase the odds of procuring viable cancer tissue from tissue sections.^9^, ^10^ However, this methodology requires the capability to process large sections of tissue (i.e. whole‐mount) and is somewhat imprecise in localising a specific region of prostate cancer tissue. It also changes the standard workflow of the pathology department, making it more challenging to implement in non‐academic settings.

We propose an alternative means of procuring tissue via 3D moulds by creating needle guides within the mould to re‐biopsy a cancerous tissue area that was previously identified on the MR‐US fusion clinical biopsy. We sought to develop and implement a novel, reliable platform for ex vivo sampling of living prostate cancer.

## 2 METHODS

### 2.1 Patient identification, inclusion criterion and information storage

**FIGURE 1:**
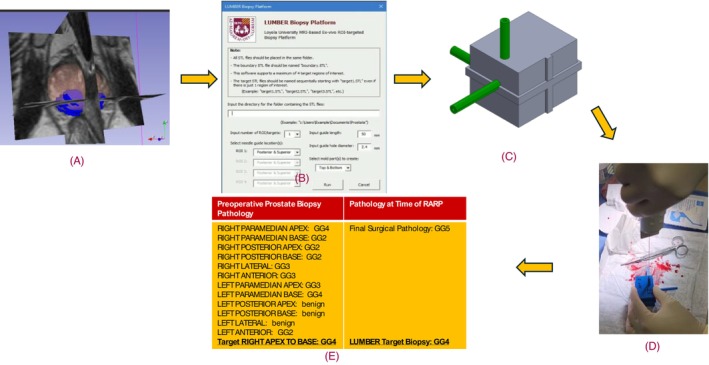
LUMBER biopsy platform sampling process for a patient: (A) 3D visualisation of prostate boundary and target lesion ROI segmentations overlaid on MR images. These segmentations are exported to STL files; (B) programme graphical user interface which imports the STL files to create mould; (C) 3D visualisation of prostate mould with needle guide; (D) ex vivo targeted sampling of prostate after prostatectomy; and (E) pathology concordance with preoperative targeted biopsy.

Study participants were identified after an MR‐US fusion transperineal prostate biopsy via the UroNav (Koninklijke Philips N. V, Netherlands) platform identified Gleason Grade Group (GG) 2 or greater prostate cancer, and patients wished to proceed with robot‐assisted radical prostatectomy (RARP). Inclusion criteria were that patients have a Prostate Imaging Reporting and Data System (PI‐RADS) score of 3 or greater on a MR‐targeted ROI (region of interest), and the PI‐RADS ≥ 3 ROIs must be positive for GG ≥ 2 on biopsy. Patients were then recruited and consented in clinic during the time of pre‐operative discussion. All patient data, including age, PSA, PSA density, magnetic resonance imaging (MRI) radiology report, biopsy report and UroNav target ROI volume, were stored in a Research Electronic data capture (REDCap) database.^11^ Figure 1 demonstrates the process from biopsy and creation of mould to procurement of living prostate tissue.

### 2.2 Creation of 3D printed prostate mould with needle guides

**FIGURE 2:**
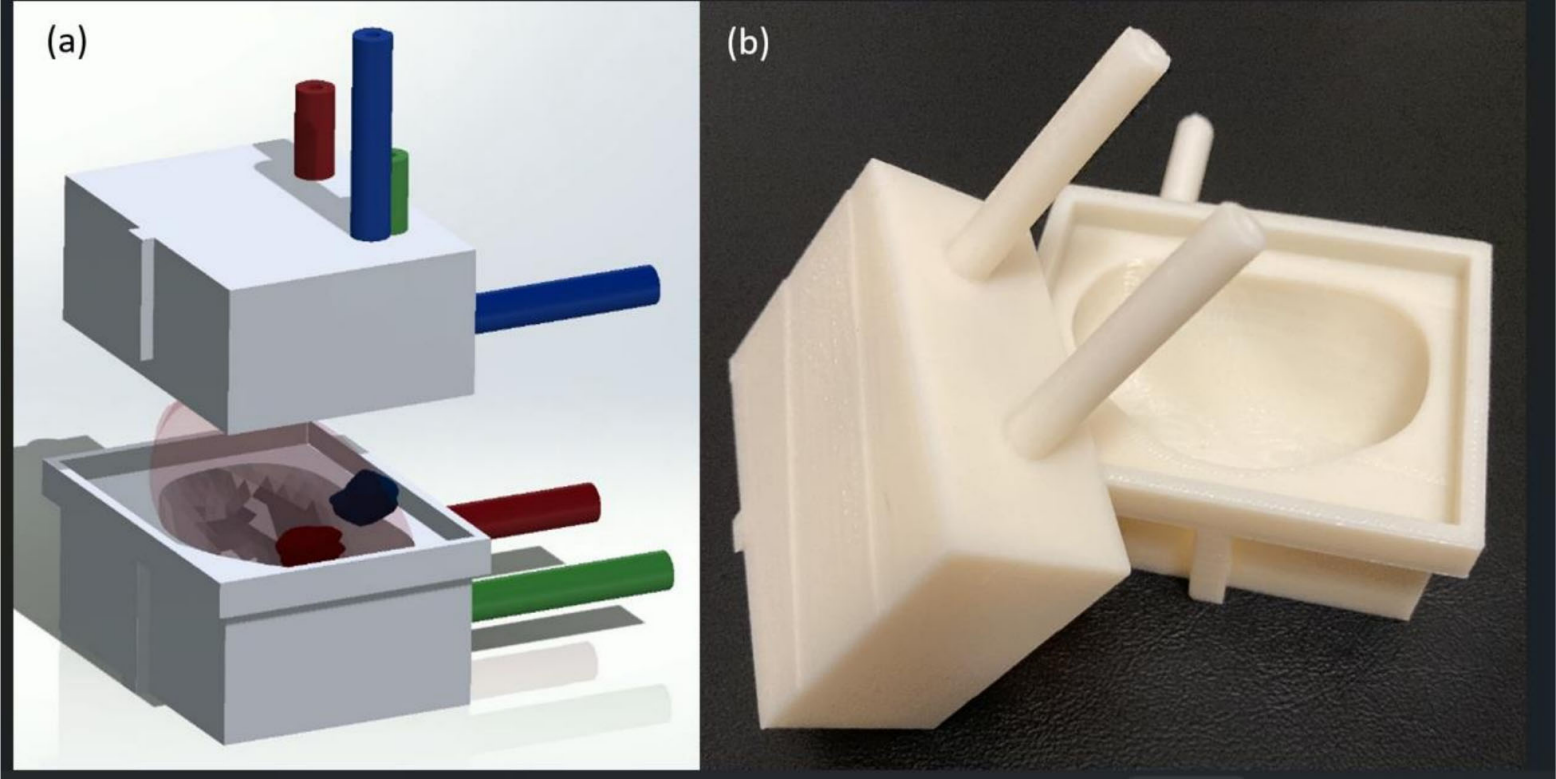
Solidworks (A) 3D model output and (B) 3D print of prostate mould with two target guides per lesion for three regions of interest.

Construction of the 3D mould with needle guides capitalises on the availability of 3D segmentations of the prostate gland and target ROIs from the clinical Radiology/Urology workflow for MR‐guided biopsies. These segmentations were exported from the UroNav system as stereolithography (STL) files, which describe the mesh surface as a series of interlocking triangles. SolidWorks (Dassault Systèmes, France), a solid modelling computer‐aided design software platform, has various tools to manipulate these 3D shapes. Through a series of sketching, extrusion, subtraction, lofting and surface thickening, 3D moulds were created around a prostate boundary with biopsy needle guides (Figure 2). Specifically, the prostate boundary was inserted into a large cube and then subtracted out to form a mould. Similarly, a centre of mass was calculated from the Radiologist‐drawn ROI, and a path was extruded to the surface of the cube to construct a needle guide for precise biopsy of that ROI. Additional manipulations were performed to optimise materials and orient the prostate within the mould.

Solidworks allows for the use of macros, which bundles together a certain sequence of inputs into a preset sequence of outputs. Macros, when programmed within Solidworks, can allow for complete automation of the complex 3D manipulations. This enables material optimisation in terms of size and wall thickness as well as customisation of biopsy needle guide number, depth, size and location. Our group created a macro for this project, called LUMBER (Loyola University MRI‐Based Ex‐vivo ROI‐targeted biopsy platform; see Figure 1b). This enabled a user with minimal experience using Solidworks to create complex, patient‐specific moulds for variable prostate morphologies and number/location of target ROIs in minutes. For each patient, three moulds were printed. The first used the original prostate boundary segmentation from MRI. The other two increased the volume of the prostate boundary surface by offsetting 1 and 2 mm, respectively. We found this enabled one mould to fit around the explanted prostate in the event of slight errors in boundary segmentation on MRI. The moulds were then 3D printed using a Fortus 250MC printer (Stratasys, Israel). Figure 2 illustrates a representative mould with multiple approaches to biopsying a positive target ROI.

### 2.3 Procurement of viable prostate cancer tissue

The 3D printed moulds were brought to the operating room at the time of RARP. Following the extraction of the prostate from the body, the seminal vesicles were removed sharply. The right seminal vesicle and the right side of the prostate were marked with clips. The prostate specimen was placed into the mould. Initially, a manual 15 cm, 14‐gauge Tru‐Cut was used because of cost compared to a firing Bard 25 cm, 18‐gauge biopsy needle. Samples were obtained through the previously created, calibrated target guides from superior, lateral and posterior approaches. The length of the target guides corresponded to the correct distance to ensure that the centre of mass of the target would be sampled by the needle according to a fixed depth of needle insertion of 5 cm. (This is defined in the GUI parameter ‘input guide length’ as shown in Figure 1b.) Mould biopsies were performed by one of three urologists (all Society of Urologic Oncology affiliated). The samples were sent for pathology alongside the prostate and seminal vesicles as one specimen.

Two experienced GU pathologists confirmed the presence and Gleason grade of carcinoma at the biopsy locations. Tissue was processed alongside the radical prostatectomy specimens as per standard of care.

### 2.4 Statistical analysis and primary outcome

This was a proof of principle study. Accuracy of the platform was evaluated by comparing the pathology results to those of the prior biopsies performed at the time of initial cancer diagnosis both in terms of presence of clinically significant prostate cancer and Gleason score concordance.

## 3 RESULTS

From 2023 to 2024, 27 patients were enrolled in the study. Twelve patients were excluded because of operator difficulties with the Tru‐Cut biopsy needle. Two patients were excluded owing to software issues with the UroNav, resulting in a non‐targetable positive ROI. One patient was excluded because of low cancer volume (~5%) on the final pathology in a 44‐ml prostate. Of note, the initial biopsy yielded two cores with 100% involvement.

The 12 patients that met the study criteria underwent RARP and utilised the LUMBER platform at time of RARP to procure fresh viable prostate cancer tissue. PSA and PSA density median and ranges were 6.38 ng/ml (4.57–14.24) and 0.18 ng/ml^2^ (0.12–0.55), respectively. Prostate volume median was 36.1 ml (25.5–48.8). PI‐RADS scores ranged from 3 to 5, and ROI volumes averaged 0.74 ml (0.14–1.74). The location of lesions included all regions of the prostate. Table 1 reports the above data for each patient included in the study.

**TABLE 1:** Cancer characteristics of patients undergoing RARP.

N	PSA	PSA density	Prostate size (cc)	PI‐RADS	Dynacad lesion volume (cc)	Lesion location	Clinical GG, biopsy	LUMBER GG, biopsy	Notes
1	9.21	0.19	48.8	5	1.74	Midgland, anterior TZ	2	4	
2	4.75	0.13	35.9	3	0.14	Midgland, posteriormedial PZ	5	2	
3	5.34	0.2	26.2	4	0.41	Midgland, posteriormedial PZ	2	1	
4	6.14	0.16	39.3	4	0.4	Apex, posteriormedial PZ	2	2	2 MRI (+) lesions, 1 not sampled on mould
5	6.62	0.12	55.4	5	1.28	Midgland, posteriormedial PZ	5	5	
6	5.24	0.14	36.2	5	1.14	Apex, posterolateral PZ	2	2	
7	4.57	0.12	39.4	5	0.84 (1); 0.50 (2)	Apex, posterolateral PZ	2	2	2 MRI (+) lesions, mould biopsies both (+)
8	9.68	0.36	27.2	4	0.96	Base, posteriomedial PZ	5	3	2 MRI lesions, only 1 (+) for cancer
9	4.90	0.14	35.6	5	0.4	Midgland, posterolateral PZ	2	4	
10	14.24	0.55	25.7	5	2.41	Apex, posteriomedial PZ	4	4	
11	14.02	0.55	25.5	5	0.63 (1); 0.13 (2)	Midgland, posterolateral PZ	2	2	2 MRI (+) lesions, smaller 0.13 cc lesion was (−) on mould biopsy
12	7.62	0.21	36.5	5	0.95	Midgland, posteriomedial PZ	3	5	2 MRI lesions, only 1 (+) for cancer

Abbreviations: GG = Gleason Grade, MRI = magnetic resonance imaging, PI‐RADS = Prostate Imaging Reporting and Data System, RARP = robot‐assisted radical prostatectomy, PZ = peripheral zone, TZ = transition zone.

All 12 (100%) patients had LUMBER biopsies positive for prostate cancer. The GG concordance rate of LUMBER biopsy to initial diagnostic biopsy was 6/12 (50%). The remaining six biopsy mismatches were split evenly between the LUMBER biopsy having a greater GG and the clinical biopsy having a greater GG. The median time from biopsy to RARP was 77 days. Two patients had multiple MRI‐positive lesions; in one patient, one lesion was very small (0.13 ml on Dynacad ROI volume), and the mould biopsy was negative.

## 4 DISCUSSION

Utilising the LUMBER platform, we were able to consistently and successfully procure viable prostate cancer tissue in 12 out of 12 patients in the study with known, MRI‐targeted positive lesions. Furthermore, there was minimal interruption to clinical workflow with limited use of additional resources. The ability to effectively and efficiently obtain living cancer tissue has the potential for advancing cell biology and molecular studies in prostate cancer.

Previously, Vitek et al. described a process to procure viable prostate tissue from non‐fixed prostatectomy specimens that were precisely sliced using a 3D printed mould with slice guide slits.^9^ At the time of sectioning, previously identified suspicious regions on MRI were localised and cognitively targeted for focal analysis. This process has two drawbacks. The first is a disruption to the standard pathology workflow, as pathology staff have to learn to use 3D moulds for slicing or to interpret and localise pre‐cut prostate specimens. The second is that cognitive targeting has limited accuracy and can be time intensive. Our method bypasses the sectioning step, thereby leaving the pathology workflow untouched. More importantly, LUMBER precisely targets areas of tissue proven to be cancerous from previous clinical biopsy procedures conducted in a similar manner.

Although 12 out of 12 specimens obtained tissue positive for prostate cancer, we note that only 6/12 (50%) had concordance with the initial lesion biopsy and 8/12 with the radical prostatectomy pathology. In studies comparing prostate biopsy pathology concordance with prostatectomy pathology, rates ranged from 28 to 76%, with discordance because of the length of the biopsy cores obtained, tumour location, pathologist misinterpretation and interobserver variability.^12^, ^13^, ^14^ Our concordance rates were similar to reported rates in literature when comparing biopsy to final pathology. With regards to LUMBER biopsy to initial biopsy variability, we hypothesise this is because of intratumor heterogeneity and hope for clarity with further development of the LUMBER platform.^15^

With regards to our excluded patients, we noted the variability of data using a Tru‐Cut 14‐gauge needle. Only one operator was able to consistently obtain data with the Tru‐Cut needle. We suspect this is from the technical aspects of a manual core biopsy needle versus a firing core biopsy needle.^16^, ^17^ The Tru‐Cut manual needle requires multiple steps to obtain a sample, in particular, a manual push–pull technique that had high operator variability in an ex vivo setting. On the contrary, using a spring‐loaded needle with fewer steps allowed for more consistent results across several operators. Since the 18 g firing biopsy needle gun is the most common instrument used in prostate biopsy, operator familiarity with the Bard biopsy needle will result in minimal workflow disruption with the LUMBER platform during radical prostatectomy.^18^

The limitations of this study are its single institution nature and the lack of testing to determine cell viability after biopsy. Nevertheless, we present a novel, ex vivo, reproducible method for procuring MRI‐visible fresh prostate cancer tissue with the potential to advance translational prostate cancer biology.

## 5 CONCLUSION

To date, there are limited methods to extract live prostate tissue for translational research. There remains much to understand regarding the tumour environment. We present a new method utilising the results from positive MR‐targeted biopsies and 3D‐printed moulds with needle guides for procuring fresh prostate tissue with minimal workflow disruption and instruments familiar to most urologists.

## AUTHOR CONTRIBUTIONS

**Chris Du:** Project design and maintenance, manuscript writing, proofreading, patient recruitment. **Goran Rac:** Project design and maintenance, manuscript writing, proofreading, project specimen handling, and patient recruitment. **Nicholas Lanzotti:** manuscript writing and patient recruitment. **Jeffrey Ellis:** Project design and maintenance, manuscript writing, proofreading and patient recruitment. **Victor Chen:** Manuscript writing and patient recruitment. **Maria M. Picken:** Project specimen handling, project design and manuscript writing. **Guliz A. Barkan:** Project specimen handling, project design and manuscript writing. **Michael Woods:** Project specimen handling, patient recruitment and manuscript writing. **Alex Gorbonos:** Project specimen handling, patient recruitment and manuscript writing. **Marcus Quek:** Project specimen handling, patient recruitment and manuscript writing. **Steven M. Shea:** Project design and maintenance, manuscript writing, proofreading, project specimen handling and patient recruitment. **Gopal N. Gupta:** Project design and maintenance, manuscript writing, proofreading, project specimen handling and patient recruitment.

## CONFLICT OF INTEREST STATEMENT

The authors declare no conflicts of interest.
